# Urban and rural variation in clustering of metabolic syndrome components in the Thai population: results from the fourth National Health Examination Survey 2009

**DOI:** 10.1186/1471-2458-11-854

**Published:** 2011-11-10

**Authors:** Wichai Aekplakorn, Pattapong Kessomboon, Rassamee Sangthong, Suwat Chariyalertsak, Panwadee Putwatana, Rungkarn Inthawong, Wannee Nitiyanant, Surasak Taneepanichskul

**Affiliations:** 1 Faculty of Medicine, Ramathibodi Hospital, Mahidol University, Rama VI Rd., Rajdevi, Bangkok 10400, Thailand; 2 Faculty of Medicine, Khon Kaen University, Khon Kaen, Thailand; 3 Epidemiology Unit, Faculty of Medicine, Prince of Songkla University, Songkhla, Thailand; 4 Faculty of Medicine, Chiang Mai University, Chiang Mai, Thailand; 5 National Health Examination Survey Office, Health System Research Institute, Nonthaburi, Thailand; 6 Faculty of Medicine Siriraj Hospital, Mahidol University, Bangkok, Thailand; 7 College of Public Health Sciences, Chulalongkorn University, Bangkok, Thailand

## Abstract

**Background:**

Information on the distribution of Metabolic syndrome (MetS) and its combinations by urban/rural areas in lower-middle income countries has been limited. It is not clear how the various combinations of MetS components varied by urban/rural population and if particular combinations of MetS are more common. This study aimed to estimate the prevalence of MetS and combinations of MetS components according to sex and urban/rural areas from a nationally representative sample of Thai adults.

**Methods:**

Data from the fourth National Health Examination Survey of 19,256 Thai adults aged 20 years and over were analyzed. MetS was defined using the harmonized criteria of six international expert groups with Asian-specific cut-point for waist circumference.

**Results:**

The prevalence of MetS was 23.2% among adults aged ≥ 20 years (19.5% in men and 26.8% in women). Among men, the prevalence of MetS in urban was higher than those in rural areas (23.1% vs 17.9%, *P *< 0.05), but among women, the prevalence was higher in rural areas (27.9% vs 24.5%, *P *< 0.05). Overall, an individual component of low high density lipoprotein (HDL) and hypertriglyceridemia were more common in rural areas, while obesity, high blood pressure and hyperglycemia were more common in urban areas. The most common combination of MetS components in men was the clustering of low HDL, hypertriglyceridemia, and high blood pressure (urban: 3.4% vs. rural: 3.9%, adjusted OR 0.9, 95%CI 0.7, 1.1). Among women, the most common combination was the clustering of obesity, low HDL, and hypertriglyceridemia (urban: 3.9% vs rural: 5.9%, adjusted OR 0.8, 95%CI 0.6, 0.9), followed by the clustering of these three components with high blood pressure (urban: 3.1% vs. rural 4.5%, adjusted OR 0.8, 95%CI 0.7, 0.9).

**Conclusion:**

Metabolic syndrome affects both urban and rural population with different pattern of MetS combinations. Dyslipidemia and obesity were the most common components among women in rural areas, hence, interventions to prevent and control these factors should be strengthened.

## Background

Metabolic syndrome (MetS), defined as a cluster of metabolic risk factors related to insulin resistance, has widely gained attention and been reported worldwide because of its association with the development of cardiovascular diseases (CVD) [1,2] and diabetes [3]. Several studies have reported on variations in the prevalence of MetS and combination of metabolic components across countries due to differences in the definition used, the characteristics and the magnitude of metabolic risk factors among the populations [4-7]. The prevalence of the clustering of MetS components was also varied by age and sex [5,8]. Certain studies have reported the higher prevalence of MetS among urban population compared to their counterparts in rural areas; however, information on the distribution of MetS and its combinations by urban/rural in lower-middle income countries has been limited [4,9]. It is not clear how the various combinations of MetS components varied by urban/rural population and if particular combinations of metabolic components are more common in urban or rural populations. Understanding the distribution of clustering of MetS components would benefit in designing more specific interventions to prevent and control the conditions for the population. Thailand, a lower-middle income country, has gone through a period of epidemic transition. Many metabolic risk factors such as obesity, diabetes and hypertension are commonly found in the urban and rural population [10-12]. Previous studies of MetS in Thailand have been reported; however, these studies have focused within specific groups of the population [13-16]. The present study aimed to determine the prevalence of MetS and the 16 combinations of the MetS risk factors by sex, and urban/rural areas in Thai population using data from the fourth National Health Examination Survey.

## Methods

### Study population

The fourth Thai National Health Examination Survey (NHES IV) 2008-2009 was a nationally representative cross-sectional survey using a multi-stage, stratified sampling of Thai adults aged 15 years and older. The survey was approved by the Ethical Review Committee for Research in Human subjects, Ministry of Public Health. The sampling method has been described elsewhere [17]. Briefly, the sampling units in each of the four stages of selection include: 1) five provinces in each of the four regions and Bangkok; 2) three to five districts in each province; 3) 13-14 electoral units (EUs) or villages from urban and rural areas, respectively; and 4) eight to ten men and women from each EU or village. The final sample size was targeted at 21,960 individuals and the final sample collected encompasses 20,450 individuals aged ≥ 15 years (93.1%). In this study, after excluding women with pregnancy at the time of survey (81 cases), a total of 19,256 adults aged 20 years and over were included in the analysis.

### Data collection

Data collection included a face to face interview conducted in the community and a subsequent health examination and blood sample collection. Data on demographic, medical history and medication were interviewed using standard questionnaires. In the field survey, a brief physical examination was performed by certified field research assistants. Three serial measurements of blood pressure were performed using automatic blood pressure monitors Microlife A100. Each subject's blood pressure was measured in a sitting position after 5 min of rest. The systolic (SBP) and diastolic (DBP) were averaged on the second and third measurement. Anthropometric measurements including weight, height, and waist circumference were performed with standard procedures. Waist circumference was measured on a horizontal plane midway level between the lower rib margin and iliac crest in cm. to the nearest 0.0 or 0.2 cm. Body mass index (BMI) was calculated as weight in kg divided by height in square meter.

Venous blood samples were obtained from participants who were asked to fast for 12 h overnight. Blood samples were then centrifuged and serum was frozen and transferred to the central laboratory center in the Faculty of Medicine, Ramathibodi hospital, Mahidol University for analysis. Plasma glucose was measured by a hexokinase enzyme method and serum total cholesterol and triglyceride were measured by enzymatic colorimetric methods. High-density lipoprotein cholesterol (HDL) was analyzed by homogeneous enzymatic colorimetric methods, using the Hitachi 917 model. The lipid test was standardized to the criteria of the Center for Disease Control and Prevention Lipid Standardization Program.

### Definition

MetS is defined according to the harmonized definition of the joint interim statement of the International Diabetes Federation Task Force on Epidemiology and Prevention, National Heart, Lung, and Blood Institute, American Heart Association, World Heart Federation, International Atherosclerosis Society and International Association for the Study of Obesity [18] as having three or more of the following five components: 1) abdominal obesity (WC): a cut-off point for Asian population at waist circumference ≥ 90 in men and ≥ 80 cm in women; 2) hypertriglyceridemia (TG): triglyceride ≥ 150 mg/dL (1.69 mmol/L) or taking lipid-lowering medication; 3) Low high-density lipoprotein (HDL) cholesterol: < 40 mg/dL (1.04 mmol/L) in men and < 50 mg/dL (1.29 mmol/L) in women; 4) high blood pressure (BP): ≥ 130/85 mmHg or treated for hypertension; 5) high fasting plasma glucose (FPG): ≥ 100 mg/dL (≥ 5.6 mmol/L) or having diabetes.

### Statistical analysis

All of the analyses were weighted to the probability of sampling and accounted for the complex survey design. Age-adjusted prevalence of each MetS components, MetS and of each of the 16 possible MetsS combinations were calculated separately for urban/rural areas.

Age-adjusted means for continuous variables and age-adjusted prevalence for dichotomous variables were calculated based on the direct method using the Thai population registry in 2008. Comparisons of difference between proportions and means were analyzed using chi-squared test and t-test, respectively. Statistical significance was set at *P *< 0.05. Logistic regression was used to examine the association of MetS and demographic variables of age, sex, area of residence (urban/rural), education levels (high school and higher, and less than high school) and smoking status (non-smoker and current smoker). Individuals who had ever smoked 100 cigarettes or more during their life time, and were currently smoking, were classified as current smokers. Alcohol consumption was assessed using graduated frequency questionnaires to quantify the amount of ethanol intake and categorized into 2 groups (low risk and medium or higher: cut-point at ≥ 41 gm/d in men and ≥ 21 gm/d in women) [19]. Leisure time physical activity assessed using global physical activity questionnaires was grouped according to those having ≥ 150 min/week and < 150 min per week [20]. The urban/rural differences in the prevalence of each clustering of MetS components were determined by logistic regressions controlling for age and education levels. All of the analyses were done using Stata software version 10.1 (Texas, USA).

## Results

The prevalence of MetS among Thai adults aged ≥ 20 years was 23.2% (95%CI 21.9, 24.5). The prevalence was higher in women than in men (26.8%, 95%CI 25.2, 28.5 vs.19.5%, 95% CI 18.0, 21.0, P < 0.05, respectively). Table 1 shows the characteristics of men and women with and without MetS in urban and rural areas. Overall, compared to those without MetS, individuals with MetS were older and more obese; had higher levels of blood pressure, fasting plasma glucose, and triglyceride, but had lower levels of HDL and lower education level. Among men with MetS, those who resided in urban had greater waist circumference, but lower triglyceride level compared to those in rural areas (*P *< 0.05). For women with MetS, the levels of the metabolic risk factors of those residing in urban and rural areas were not significantly different except for obesity (Table 1).

**Table 1:** Age-adjusted Mean (SE) and percentage (SE) of selected characteristics of Thai adults aged ≥ 20 years with and without metabolic syndrome, NHES IV 2009

	Men				Women			
	**Urban**		**Rural**		**Urban**		**Rural**	

	**Absent (n = 3414)**	**Present (n = 1404)**	**Absent (n = 3398)**	**Present (n = 937)**	**Absent (n = 3604)**	**Present (n = 2031)**	**Absent (n = 2888)**	**Present (n = 1580)**

Age (yr)	45.6 (0.6)	51.0 (0.6)^c^	44.0 (0.2)	49.3 (0.4)^d^	45.1 (0.4)	54.1 (0.3)^b, c^	43.1 (0.2)	52.1 (0.3)^b, d^

SBP (mmHg)	122.6 (0.4)	135.7 (1.0)^c^	121.0 (0.4)	133.7 (0.8)^d^	115.8 (0.3)	128.6 (0.6)^c^	115.8 (0.3)	129.7 (0.4)^d^

DBP (mmHg)	76.9 (0.3)	85.7 (0.8)^a, c^	74.6 (0.3)	82.9 (0.5)^a, d^	71.7 (0.2)	78.6 (0.4)^c^	71.6 (0.2)	79.8 (0.3)^d^

BMI (kg/m^2^)	23.0 (0.1)	28.8 (0.3)	22.1 (0.1)	28.0 (0.2)	23.8 (0.1)	28.9 (0.2)^b^	23.0 (0.1)	27.4 (0.2)^b^

Waist (cm)	80.0 (0.3)	96.1 (0.6)^a, c^	76.3 (0.2)	92.7 (0.7)^a, d^	77.2 (0.4)	90.0 (0.4)^b, c^	75.4 (0.3)	87.1 (0.3)^b, d^

FPG (mg/dL)	87.5 (0.9)	109.1 (3.0)	85.9 (0.8)	106.3 (2.7)	84.4 (1.0)	105.1 (2.1)	84.7 (0.7)	99.4 (1.6)

HDL (mg/dL)	48.9 (0.5)	39.3 (0.4)^a, c^	46.6 (0.4)	36.6 (0.4)^a, d^	53.9 (0.3)	41.9 (0.4)^c^	50.0 (0.5)	42.3 (0.4)^d^

Triglyceride (mg/dL)	137.1 (2.5)	257.5 (5.7)^c^	148.4 (3.4)	282.5 (6.3)^d^	104.8 (2.1)	194.3 (2.6)^c^	117.8 (1.6)	207.0 (6.5)^d^

Educational level (%)								

< high school	58.0 (1.7)	61.7 (2.0)^a, c^	78.7 (1.1)	77.4 (2.3)^a, d^	61.9 (1.0)	76.7 (2.0)^b, c^	79.5 (1.0)	88.4 (1.4)^b, d^

Leisure time physical activity (min/week) (%)								

< 150	67.2 (1.2)	73.9 (1.8)	73.7 (1.1)	70.6 (2.1)^d^	79.5 (0.8)	79.7 (2.09)	82.9 (0.7)	81.2 (1.7)^d^

Regular smoking (%)								

Yes	35.2 (1.0)	30. 4 (2.6)^c^	45.0 (1.3)	40.8 (2.6)^d^	2.6 (0.3)	4.9 (1.5)^b, c^	2.2 (0.2)	1.0 (0.1)^b, d^

Alcohol drinking (%)								

≥ 41 gm/d in men and ≥ 21 gm/d in women	15.6 (0.7)	18.9 (1.7)^c^	13.9 (0.8)	13.9 (2.0)^d^	2.4 (0.3)	1.5 (0.3)^c^	1.4 (0.2)	0.9 (0.3)^d^

*Age-adjusted: direct adjustment using Thai registered population 2008

^a ^Statistically significant difference between men in urban and rural areas at P < 0.05

^b ^Statistically significant difference between women in urban and rural areas at P < 0.05

^c ^Statistically significant difference between men and women in urban area at P < 0.05

^d ^Statistically significant difference between men and women in rural area at P < 0.05

Table 2 shows the age-adjusted prevalence of each metabolic risk factor by sex and urban/rural areas. Of all participants, hypertriglyceridemia was the most common component of MetS in men (42.9%), whereas low HDL was the most common feature in women (58.1%), followed by abdominal obesity (45.4%). Men had higher prevalence of hypertriglyceridemia (*P *< 0.05) and high blood pressure (*P *< 0.05) than women, but women had higher prevalence of abdominal obesity (*P *< 0.05) and low HDL (*P *< 0.05) than men. Prevalence of high FPG was relatively similar by sex. In general, the age-specific prevalence of each component increased with age and declined in the older age group except for low HDL and high blood pressure. There were differences in the prevalence of obesity, hypertriglyceridemia and low HDL by urban/rural areas for both sexes. Residents in urban areas had a higher prevalence of abdominal obesity (*P *< 0.05), but had a lower prevalence of hypertriglyceridemia and low HDL compared to their counterparts in rural areas, in both sexes (all *P *< 0.05). Urban men also had a higher prevalence of high FPG and high blood pressure than rural men (all *P *< 0.05), but the prevalence of high blood pressure and high FPG were not significantly different between urban and rural women.

**Table 2:** Age-adjusted prevalence of abnormal metabolic component among Thai adults aged ≥ 20 years, NHES IV 2009

	No. of participants	Abdominal obesity	Hypertriglyceridemia	Low HDL	High blood pressure	High FPG or diabetes
Men						

Urban	4818	28.6 (1.5)^a^	41.7(1.4)^a^	30.0 (1.0)^a^	41.4 (1.3)^a^	20.5 (1.8)^a^

Rural	4335	15.2 (0.8)	43.4 (1.9)	36.3 (1.2)	34.0 (1.6)	15.7 (1.0)

Age group						

20-29	764	15.0 (1.2)	32.8 (2.1)	25.1 (1.8)	22.7 (1.8)	5.9 (0.8)

30-39	1144	17.0 (1.2)	45.7 (2.1)	32.7 (1.2)	25.6 (1.6)	12.3 (1.3)

40-49	1431	20.0 (1.3)	49.6 (2.4)	39.1 (1.7)	38.9 (1.7)	20.2 (1.7)

50-59	1308	24.9 (1.1)	48.1 (2.0)	38.1 (1.9)	47.3 (1.8)	26.5 (1.5)

60-69	2498	23.4 (2.2)	41.4 (1.8)	39.1 (1.2)	58.3 (1.9)	29.0 (1.1)

70-79	1566	20.4 (1.9)	35.5 (1.5)	41.1 (2.7)	63.5 (2.1)	30.5 (1.5)

80+	442	14.0 (1.4)	29.2 (2.1)	49.0 (2.1)	67.5 (2.4)	29.8 (2.4)

All	9153	19.1 (1.0)	42.9 (1.6)	34.4 (1.0)	36.4 (1.4)	17.2 (1.1)

Women						

Urban	5635	48.2 (1.2)	25.1 (1.0)^a^	48.5 (1.0)^a^	31.0(0.7)	17.4 (1.1)

Rural	4468	43.4 (1.4)	33.9 (1.3)	63.0 (1.5)	29.4 (0.7)	15.4 (0.8)

Age group						

20-29	737	33.6 (2.0)	19.7 (1.4)	56.5 (1.7)	4.7 (0.7)	3.0 (0.5)

30-39	1340	39.8 (1.9)	25.3 (1.4)	58.7 (1.4)	16.4 (0.9)	7.6 (0.8)

40-49	1799	51.0 (1.6)	29.6 (1.3)	56.4 (1.3)	31.3 (1.1)	15.5 (1.1)

50-59	1523	57.6 (1.4)	42.9 (2.3)	58.4 (2.3)	49.3 (1.2)	30.3 (1.4)

60-69	2559	53.5 (1.5)	46.4 (1.4)	62.4 (1.7)	58.1 (1.6)	32.9 (1.5)

70-79	1652	45.7 (1.7)	46.7 (1.6)	69.1 (1.6)	65.7 (1.4)	32.1 (1.2)

80+	493	29.7 (2.0)	38.5 (2.2)	68.3(2.3)	68.6 (2.4)	25.7 (2.0)

All	10103	45.0 (1.2)	31.2(1.1)	58.7 (1.2)	30.0 (0.6)	16.0 (0.7)

*Age-adjusted: direct adjustment using Thai registered population 2008

^a ^Statistically significant difference between urban and rural areas of the same sex at P < 0.05

Table 3 shows the age-adjusted prevalence of abnormal metabolic factors by number of abnormalities. About one-fourth of the population had no metabolic abnormalities. Among men, the prevalence of having one metabolic abnormality was significantly higher in rural than in urban areas, but the prevalence of three and five metabolic abnormalities were significantly higher in urban than in rural areas. Among women, the prevalence of having two and three abnormal metabolic factors were significantly higher among rural women (Table 3).

**Table 3:** Age-adjusted prevalence (SE) of abnormal metabolic components by number of abnormalities in Thai adults aged ≥ 20 years, NHES IV 2009

		No. of abnormal metabolic factors						
	**N**	**0**	**1**	**2**	**3**	**4**	**5**	**≥ 3**

Men								

Urban	4818	26.1 (1.1)	26.7 (1.1)^a^	24.1 (0.8)	14.9 (0.5)^a^	6.1 (0.4)	2.1 (0.2)^a^	23.1(0.9)^a^

Rural	4335	26.8 (0.9)	33.1 (0.7)	22.1 (0.7)	11.5 (0.5)	5.2 (0.4)	1.2 (0.2)	17.9(0.8)

Age group								

20-29	764	41.6 (1.4)	32.7 (1.5)	16.4 (1.2)	6.5 (0.7)	2.2 (0.6)	0.4 (0.2)	9.2(0.8)

30-39	1144	27.2 (1.3)	38.8 (1.6)	20.0 (1.1)	9.6 (0.7)	3.3 (0.5)	1.1 (0.4)	14.1(1.0)

40-49	1431	22.5 (1.3)	28.4 (1.0)	25.8 (1.7)	13.9 (1.0)	7.8 (0.7)	1.4 (0.3)	23.2(1.5)

50-59	1308	20.1 (1.1)	24.7 (1.2)	25.4 (1.0)	19.3 (1.4)	7.7 (0.6)	2.8 (0.3)	29.8(1.5)

60-69	2498	15.8 (0.9)	26.1 (1.1)	28.2 (1.1)	19.1 (1.1)	8.5 (0.5)	2.4 (0.3)	29.9(1.6)

70-79	1566	12.0 (1.0)	31.7 (1.5)	29.1 (0.7)	16.7 (1.0)	7.5 (0.7)	3.0 (0.5)	27.2(1.4)

80+	442	12.1 (1.3)	30.2 (2.0)	31.3 (2.2)	15.2 (1.3)	8.6 (1.1)	2.6 (0.8)	26.4(2.0)

All	9153	26.5 (0.7)	31.3 (0.6)	22.6(0.6)	12.5 (0.4)	5.5 (0.3)	1.5 (0.2)	19.5(0.7)

Women								

Urban	5635	24.4 (0.6)^a^	29.4 (0.7)	21.6 (0.7)^a^	14.1 (0.3)^a^	7.7 (0.2)	2.7 (0.2)	24.5(0.6)^a^

Rural	4468	19.0 (0.8)	28.0 (0.8)	25.3 (0.8)	16.7 (0.7)	8.6 (0.4)	2.3 (0.2)	27.7(1.0)

Age group								

20-29	737	35.5 (1.8)	33.2 (1.4)	22.3(1.5)	7.2 (1.1)	1.7 (0.4)	0	8.9(1.2)

30-39	1340	24.7 (1.1)	33.6 (1.1)	25.8 (1.0)	11.4 (1.1)	4.2 (0.6)	0.4 (0.1)	16.0(1.2)

40-49	1799	18.5 (0.9)	28.7 (1.0)	25.6 (1.0)	17.0 (1.2)	8.6 (0.6)	1.6 (0.3)	27.2(1.2)

50-59	1523	11.2 (0.9)	20.8 (1.2)	22.9 (1.0)	23.5 (1.0)	15.6 (1.2)	6.0 (0.7)	45.1(1.7)

60-69	2559	8.2 (0.5)	19.9 (0.9)	24.8 (0.9)	23.2 (0.9)	16.3 (0.8)	7.6 (0.6)	47.1(1.2)

70-79	1652	5.0 (0.5)	19.7 (1.0)	27.3 (0.8)	27.0 (1.0)	15.3 (0.7)	5.7 (0.5)	48.0(1.3)

80+	493	6.3 (0.9)	29.6 (2.0)	23.5 (1.8)	27.4 (1.6)	10.7 (1.3)	2.6 (0.6)	40.7(2.1)

All	10103	20.6 (0.7)	28.4 (0.6)	24.3(0.6)	15.9 (0.5)	8.3 (0.3)	2.4 (0.1)	26.7(0.8)

*Age-adjusted: direct adjustment using Thai registered population 2008

^a ^statistically significant difference between urban and rural areas of the same sex at P < 0.05

Table 4 shows factors that were associated with the prevalence of MetS in men and women. These factors included age, urbanization, educational level, alcohol consumption and BMI; however, the magnitude of association varied by sex. After controlling for potential confounding factors in the multivariable analysis, it appears that urbanization was negatively associated with MetS in women, but not in men. Women who attained less than high school education had an additional 60% risk of MetS compared to those having education of high school or higher. Alcohol consumption was associated with Mets in men but not in women. BMI was highly associated with MetS in both sexes. No significant association between leisure time physical activity and MetS was observed.

**Table 4:** Adjusted Odds Ratio (OR, 95%CI) of factors that associated with metabolic syndrome in Thai adults aged ≥ 20 years, NHES IV 2009

	Male		Women	
	
	Age-adjusted^a ^(95%CI)	Multivariable-adjusted^b ^(95%CI)	Age-adjusted^a ^(95%CI)	Multivariable-adjusted^b ^(95%CI)
Age (yr)	1.03 (1.02, 1.03)	1.05 (1.04, 1.06)	1.05 (1.04, 1.05)	1.06 (1.05, 1.06)

Area of residence				

Rural	1	1	1	1

Urban	1.33 (1.17, 1.58)	0.92 (0.79, 1.08)	0.84(0.74, 0.95)	0.70(0.61, 0.80)

Education level				

High school and higher	1	1	1	1

< high school	0.93 (0.80, 1.09)	1.21 (1.03, 1.41)	2.07 (1.75, 2.44)	1.60 (1.33, 1.93)

Smoking				

No	1	1	1	1

Yes	0.71 (0.62, 0.82)	1.13 (0.97, 1.33)	0.71 (0.47, 1.07)	1.05 (0.62, 1.79)

Alcohol drinking				

< 40 gm/d in men, < 20 gm/d in women	1	1	1	1

≥ 40 gm/d men, ≥ 20 gm/d in women	1.20 (0.96, 1.50)	1.52 (1.19, 1.94)	0.89 (0.57, 1.39)	1.02 (0.69, 1.51)

Leisure time physical activity (min.)				

≥ 150	1	1	1	1

< 150	1.09 (0.94, 1.25)	1.16 (0.98, 1.37)	0.92 (0.82, 1.03)	0.92 (0.79, 1.06)

BMI (kg/m^2^)	1.46 (1.43, 1.50)	1.48 (1.44, 1.51)	1.26 (1.23, 1.29)	1.26 (1.23, 1.29)

^a^Age-adjusted model

^b^Multivariable-adjusted model controlling for age, education level, current smoking, alcohol drinking, leisure time physical activity and BMI

Table 5 shows the age-adjusted prevalence and proportions of all possible MetS combinations by sex and urban/rural areas. The prevalence of the all possible combinations of MetS components ranged from 0.2 to 3.9% in men and 0.2 to 5.9% in women. The most common combination was the clustering of low HDL, high blood pressure and hypertriglyceridemia of 3.7% in men (3.4% in urban vs 3.9% in rural areas, *P *= 0.35) and the clustering of obesity, low HDL and hypertriglyceridemia of 5.2% in women (3.9% in urban vs 5.9 in rural areas, *P *< 0.05). For the MetS combination of all five components, the prevalence was higher in urban areas than in rural areas in both sexes (all *P *< 0.05). After adjusted for age and educational level, of the 16 possible combinations in men, eight of which were similarly found in urban and rural areas and seven of which were significantly more common in urban areas than in rural areas leaving only the clustering of low HDL, hypertriglyceridemia and high FPG that was less common in urban compared to rural areas (OR 0.5, 95%CI 0.3, 0.9). Among women, nine of the combinations were not significantly different between urban and rural areas and four combinations were more common in urban areas. There were three combinations which comprised the clustering of dyslipidemia (low HDL, and hypertriglyceridemia) with another factor of obesity (OR 0.8, 95%CI 0.6, 0.9) or with high blood pressure (OR 0.5, 95%CI 0.4, 0.7) or both (OR, 0.8, 95%CI 0.7, 0.9) which were less common in urban compared to rural areas.

**Table 5:** Age-adjusted prevalence, proportions and adjusted prevalence odds ratio (OR) for metabolic syndrome by metabolic combination among Thai adults aged ≥ 20 years, NHES IV 2009

	Men (*n *= 9,153)					Women (*n *= 10,103)				
	**Urban**		**Rural**			**Urban**		**Rural**		

	**Prevalence (%)**	**Proportion (%) among MetS**	**Prevalence (%)**	**Proportion (%) among MetS**	**OR (95%CI)**	**Prevalence (%)**	**Proportion (%) among MetS**	**Prevalence (%)**	**Proportion (%) among MetS**	**OR (95%CI)**

WC+HDL+BP	0.8	3.1	0.5	2.3	1.9 (1.1, 3.4)^a^	3.2	13.9	3.0	10.5	1.2 (0.9, 1.5)

WC+HDL+TG	2.7	14.4	2.3	16.2	0.9 (0.6, 1.4)	3.9	23.3	5.9	29.3	0.8 (0.6, 0.9)^b^

WC+HDL+FG	0.2	0.8	0.2	1.1	0.8 (0.4, 1.7)	1.4	7.2	1.1	4.7	1.6 (1.1, 2.2)^a^

WC+BP+TG	2.3	10.2	1.3	7.3	1.7 (1.0, 2.9)^a^	1.0	2.7	1.0	3.5	1.2 (0.9, 1.6)

WC+BP+FG	2.5	9.0	0.6	2.3	4.7 (2.9, 7.7)^a^	2.4	7.1	1.8	5.0	1.5 (1.2, 1.8)^a^

WC+TG+FG	0.5	2.3	0.2	1.3	3.0 (1.1, 8.4)^a^	0.2	0.9	0.4	2.1	0.7 (0.3, 1.8)

HDL+BP+TG	3.4	14.5	3.9	22.1	0.9 (0.7, 1.1)	1.3	3.9	2.5	7.6	0.5 (0.4, 0.7)^b^

HDL+BP+FG	1.0	3.4	0.9	4.0	1.2 (0.8, 1.7)	0.5	1.4	0.5	1.1	0.9 (0.6, 1.6)

HDL+TG+FG	0.7	3.0	1.2	5.9	0.5 (0.3, 0.9)^b^	0.2	0.6	0.5	1.5	0.6 (0.2, 1.2)

BP+TG+FG	1.6	6.2	0.9	4.0	1.9 (1.2, 2.9)^a^	0.1	0.3	0.2	0.5	0.5 (0.3, 1.0)

WC+HDL+BP+TG	2.5	11.0	2.3	14.1	1.2 (0.8, 1.6)	3.1	13.5	4.5	16.4	0.8 (0.7, 0.9)^b^

WC+HDL+BP+FG	1.1	4.1	0.5	2.3	2.2 (1.4, 3.6)^a^	1.5	3.9	1.1	3.4	1.4 (1.0, 2.0)^a^

WC+HDL+TG+FG	0.7	3.8	0.5	2.3	1.0 (0.5, 1.7)	1.6	8.6	1.4	3.9	1.2 (0.9, 1.6)

WC+BP+TG+FG	1.1	4.3	0.8	3.9	1.3 (0.9, 2.1)	1.0	3.3	0.9	2.8	1.2 (0.9, 1.7)

HDL+BP+TG+FG	0.7	2.4	1.1	4.5	0.6 (0.4, 1.1)	0.6	1.4	0.8	2.1	0.8 (0.6, 1.2)

WC+HDL+BP+TG+FG	2.1	7.4	1.2	6.7	2.0 (1.4, 2.7)^a^	2.7	8.1	2.4	5.5	1.4 (1.1, 1.7)^a^

Age-adjusted prevalence and proportion: direct adjustment using the Thai registered population 2008; OR: Odds ratio for metabolic syndrome (urban = 1, rural = 0) adjusted for age and education level; prevalence were calculated among the population, proportions were calculated among those having metabolic syndrome; ^a ^urban have significantly higher prevalence compared to rural areas, ^b ^rural have significantly higher prevalence compared to urban areas; WC: abdominal obesity (waist circumference ≥ 90 cm. in men and ≥ 80 cm. in women); HDL, low high-density lipoprotein-cholesterol (< 40 mg/dL in men and < 50 mg/dL in women); BP, high blood pressure (≥ 130/85 mmHg or hypertension); FG, hyperglycemia (fasting plasma glucose ≥ 100 mg/dL or diabetes)

## Discussion

The findings from this study have shown that one-fourth, an estimated 12 million, of the Thai adults aged ≥ 20 years had MetS. The prevalence was higher in women than in men and it was higher in rural women than in urban women. The most common abnormal metabolic risk factor was dyslipidemia (hypertriglyceridemia in men and low HDL in women). The clustering of three components comprising dyslipidemia (low HDL and hypertriglyceridemia) and another factor tended to be higher in the rural compared to urban population. Previous studies have reported varied prevalence of MetS according to the population under study [13-15]. The prevalence of MetS found in this study was slightly lower than that of some Asian and the US population [5-7]. The study in Korea using similar criteria for waist circumference but a higher FPG cut-point reported a prevalence of MetS of 28% in women and 24.6% in men [6]. The study in Shanghai, China reported the prevalence of 35% in women and 28% in men [7], whereas the prevalence in the US, with a higher cut-point for abdominal obesity, were 32.4% in women and 36.1% in men [5]. The finding of a higher prevalence of MetS among women compared to men seems to be unique for Asians, but it is in contrast with the findings from the US [5]. The significantly higher prevalence of obesity, and low-HDL among women compared to men could explain the higher prevalence of MetS among women in the Thai population.

An interesting finding in the present study is that the prevalence of MetS in rural women was higher than that of urban women. This pattern was inconsistent with findings from studies in India and China [4,9,20], where the prevalence of MetS were higher in urban populations. The higher prevalence of MetS in the rural women was largely attributed to the higher prevalence of hypertriglyceridemia and low HDL with another component among people in rural areas. Compared to other countries, the prevalence of hypertriglyceridemia in the present study was rather similar to that of China (40%) [20], but higher than that of Korea (32.6%) [6] and the US study (31.4%) [5]. For low HDL, the prevalence in the Thai population was relatively similar to that of Korea but higher than that of the Chinese (19.3%) and the US. population (25.4%). With regard to the prevalence of hyperglycemia and abdominal obesity in the present study, both were lower than those of the Western countries [5,8,21,22], but were relatively similar to other Asian populations [6,7,23].

This study revealed that the most common metabolic cluster was different between sexes, e.g., the clustering of low HDL, high blood pressure and hypertriglyceridemia in men, and the clustering of obesity, low HDL and hypertriglyceridemia in women. This pattern is similar to that of some Asian populations, but rather different from the western populations where abdominal obesity was the most common factor in both sexes [5,6,23]. The higher prevalence of dyslipidemia among rural populations is likely to be due to the difference in dietary patterns where people consume higher proportions of carbohydrates [24]. A study by Mckeown reported that dietary glycemic index and carbohydrates were positively associated with level of fasting triglyceride but inversely associated with HDL [25]. The higher rate of low HDL in rural people might be also partly due to the higher rates of smoking in rural men than in urban men [24]. For women, the high prevalence of low HDL might also be attributed to obesity and a sedentary life style.

Previous studies have reported the variable associations between the various clustering of MetS components and health outcomes [2,26]. It is not completely clear whether different combinations play different degrees of risk to the outcome. However, some studies indicated that the combination of five components and four components without hypertriglyceridemia confer the highest risk to CVD and all-cause mortality [2,8]. This might suggest that despite the relative similar prevalence of MetS among people in urban and rural areas, they might not be carrying the same risk. With the higher prevalence of combinations with five components and other factors containing high blood pressure and hyperglycemia in urban populations, it possible that, on average, those residing in the urban areas might still be at a greater risk of CVD outcomes compared to those in rural areas. Finally, the findings that education was inversely associated with MetS, particular in women, was consistent with other studies in the US and China (5, 20). Lower education might contribute to limited nutritional knowledge and inappropriate food choice [27].

The implication of this study is that the findings of highest prevalence of dyslipidemia in the rural areas suggest that intervention in rural areas should target more on the detection and treatment of the conditions. Of note, the existing facilities for testing of lipid profile were available in general hospitals but not in primary care settings, where the delivery of health care largely takes place, as a previous study indicated that the rates of detection, treatment and control of dyslipidemia were markedly suboptimal [28].

There were some limitations in this study. First, the cross-sectional design precludes the interpretation of causal relationship between independent risk factors and the development of MetS. Second, there is no data about the treatment of hypertriglyceridemia and low HDL; however, it is likely that the detection and treatment rates were low since the laboratory measurement of serum triglyceride and HDL were not usually done in the general population. In this case, it is not likely to affect the estimated prevalence. Despite the limitations, this study has strength in its large sample size with a national representative sampling to estimate the prevalence at a national level.

## Conclusions

The present study indicates that MetS is very common in the Thai population and the syndrome affects the whole population with greater proportion among women in rural areas and those with a low level of education. This information should prompt awareness that this condition is no longer confined to urban but rather in rural areas as well. As dyslipidemia and obesity were very common, preventive measures to promote a healthy diet and physical activity, particular in rural women and those with low education, in order to avoid the unnecessary burden from CVD in the future are critical.

## Abbreviations

MetS: Metabolic syndrome; NHES: National Health Examination Survey; EU: Electoral unit; HDL: High-density lipoprotein cholesterol; WC: Abdominal obesity (waist circumference ≥ 90 cm. in men and ≥ 80 cm. in women); BP: High blood pressure; TG: Hypertriglyceridemia; FG: Hyperglycemia; BMI: Body mass index (mg/kg2); mmol/L: Millimol per litre; mg/dL: Milligram per deciliter; SBP: Systolic blood pressure; DBP: Diastolic blood pressure

## Competing interests

The authors declare that they have no competing interests.

## Authors' contributions

WA designed and managed the project, analysed data and wrote the manuscript. PK collected data and reviewed/edited the manuscript. RS collected data and reviewed/edited the manuscript. SJ collected data and reviewed/edited the manuscript. PP collected data and reviewed/edited the manuscript. WN reviewed/edited the manuscript. ST collected data and reviewed/edited the manuscript. All the authors participated in approving the final draft of the manuscript.

## Pre-publication history

The pre-publication history for this paper can be accessed here:

http://www.biomedcentral.com/1471-2458/11/854/prepub
